# Prognostic value of monocyte and neutrophils to lymphocytes ratio in patients with metastatic soft tissue sarcoma

**DOI:** 10.18632/oncotarget.3283

**Published:** 2015-03-20

**Authors:** Long Jiang, Shanshan Jiang, Dongrong Situ, Yongbin Lin, Han Yang, Yuanfang Li, Hao Long, Zhiwei Zhou

**Affiliations:** ^1^ Sun Yat-sen University Cancer Center, Guangzhou, China; ^2^ Collaborative Innovation Center for Cancer Medicine, Guangzhou, China; ^3^ State Key Laboratory of Oncology in South China, Guangzhou, China; ^4^ University of California, San Francisco, CA, USA

**Keywords:** soft tissue sarcoma, metastasis, immunity, prognosis

## Abstract

Metastatic soft tissue sarcomas (STS) represent enormous challenges to improve the low survival rate, which is almost the same as past 2 decades ago. Prognosis of cancer patients are based not only on tumor-related factors but also on host-related factors, particularly systemic inflammatory response. We evaluated the association among possible risk factors and survival for metastatic STS by reviewed a single-institution nearly 50-year experience. We found that both monocyte ratio and NLR ratio were significant prognostic predictors for OS and PFS of metastatic STS. And patients with monocyte ratio or NLR ratio > 1 should be screened out as candidates for more intensive or aggressive multimodality treatments and more aggressive follow-up. For this reason, this result could serve as a basis for future prospective study.

## INTRODUCTION

Soft tissue sarcomas (STS), arising from almost any embryonic mesodermal tissue, account for nearly 1% of newly diagnosed malignancies annually [1]. Under multimodality treatment, patients with localized disease have estimated 5-year survival rates of about 70% [2–4]. However, metastatic STS, constituting approximately 40% of metastatic osteosarcoma and also the second most common cause of pulmonary metastatic disease, still represent enormous challenges to improve the low survival rate [5, 6]. Despite advances in chemotherapy, radiation therapy and surgery, the three year survival of patients with metastatic sarcoma is 20–45%, which is almost the same as past 2 decades ago [6–9].

Nowadays, increasing data indicated that prognosis of cancer patients are based not only on tumor-related factors but also on host-related factors, particularly systemic inflammatory response [10, 11]. As a significant indicator patients’ inflammation status, neutrophil/lymphocyte ratio (NLR) was proved as an predictor of prognosis in colon cancer, lung cancer, and liver cancer [12–16]. Increased counts of neutrophils and/or decreased counts of lymphocytes might serve as suppressor of lymphokine-activated killer cells, which could increase the propensity to metastasis [17]. Recent studies have illustrated that circulating monocyte count could be an independent risk factor of poor prognosis in various cancers [18, 19].

Our aim of this study is to determine whether possible risk factors (age, sex, size of primary tumor, tumor depth, pathological subtypes, pathological grade, monocyte ratio and NLR ratio) were associated with survival for metastatic STS.

## RESULTS

142 of 154 patients with metastatic STS were eligible for the final analysis. In this group of 142 patients, the mean age was 44.35 years (range: 5–71 years, median 47.5 years); 60 patients were male (42.3%) and 82 female (57.7%). The tumors pathological subtypes included so-called fibrohistiocytic tumors in 22 patients (15.5%), undifferentiated sarcomas in 96 (67.6%), smooth muscle tumors in 22 (15.5%), and fibroblastic/myofibroblastic tumors in 2 (1.4%) (Table 1).

**Table 1:** Clinicopathological correlation of monocyte ratio and NLR ratio and in Patients with Metastatic STS

Characteristic	All (*n* = 142)		monocyte ratio <= 1 (*n* = 99)	monocyte ratio > 1 (*n* = 43)	*P*	NLR ratio <= 1 (*n* = 89)	NLR ratio > 1 (*n* = 53)	*P*
Age, yrs	47.5^†^ (range: 5–71)		47^†^ (range: 6–70)	49^†^ (range: 5–71)	0.428	43^†^ (range: 6–70)	50^†^ (range: 5–71)	0.368
Sex (%)					0.498			0.246
Male	60	42.30%	40	20		35	25	
Female	82	57.70%	59	23		54	28	
Primary Tumor Size (cm)	5.5^†^ (range: 0.5–20)		5.75^†^ (range: 0.5–18)	5.4^†^ (range: 1–20)	0.799	5.5^†^ (range: 0.5–20)	5.75^†^ (range: 1–17.4)	0.249
Primary Tumor Depth (%)					0.601			0.363
Superficial	44	31%	32	12		30	14	
Deep	98	69%	67	31		59	39	
Pathological Subtypes (%)					0.42			0.736
So-called Fibrohistiocytic Tumors	22	15.50%	17	5		14	8	
Undifferentiated Sarcomas	96	67.60%	63	33		59	37	
Smooth Muscle Tumors	22	15.50%	17	5		14	8	
Fibroblastic/Myofibroblastic Tumors	2	1.40%	2	0		2	0	
Pathological Grade (%)					0.129			0.135
1	9	6.30%	4	5		4	5	
2	13	9.20%	11	2		11	2	
3	120	84.50%	84	36		74	46	
Follow-up (months)					0.82			0.45
Median	49.38		50.83	47.37		47.56	51.2	
Range	2.97–476.17		3.04–476.17	2.97–468.9		2.97–476.24	3.21–476.17	
Mean	71.05		71.65	69.67		69.8	73.15	

† Median values are listed

The mean follow-up for survivors as of December 2014 was 71.05 months (range: 2.97–476.17 months, median 49.38 months). The mean tumor size at diagnosis was 6.68 cm (range 0.5–20 cm, median 5.5 cm).

**Figure 1:**
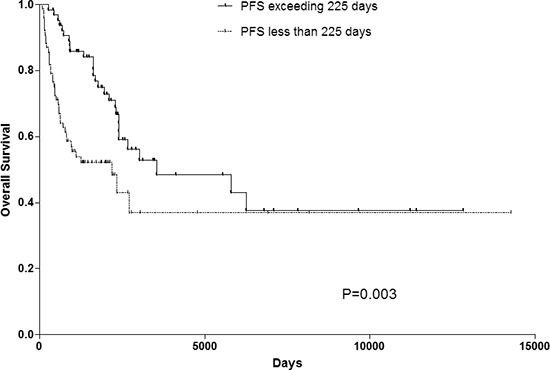
OS rate of patients with PFS exceeding 225 days or not. PFS progress-free survival, OS overall survival

**Figure 2:**
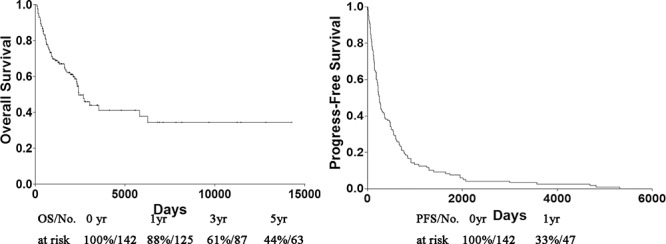
OS and PFS of patients with metastatic STS. PFS progress-free survival, OS overall survival

Univariate analysis showed a PFS exceeding 225 days (*p* = 0.003) as prognostic factors (Fig. 1). Age (*P* = 0.238), Sex (*P* = 0.783), size of primary tumor (*P* = 0.425), tumor depth (*P* = 0.484), pathological subtypes (*P* = 0.861) and pathological grade (*P* = 0.965) did not have any significant impact on OS (Table 2). Median OS was 2411 days and 28.2% of the patients were alive without disease, 25.4% were alive with disease, 45.8% dies of disease, while 0.7% (1 patients) died from other causes (heart disease). The overall 1-, 3- and 5-year OS rates were 88%, 61% and 44% each, respectively (Fig. 2).

**Table 2:** Univariate analysis of prognostic factors for OS and PFS in patients with Metastatic STS

Variables	OS			PFS		
	HR	95%CI	*P* value	HR	95%CI	*P* value
Age	1.010	0.993–1.027	0.238	0.990	0.977–1.002	0.107
Sex	0.932	0.562–1.543	0.783	1.298	0.909–1.852	0.150
Size of Primary Tumor	1.003	0.996–1.009	0.425	1.001	0.995–1.006	0.777
Tumor Depth	1.211	0.708–2.069	0.484	0.736	0.504–1.075	0.120
Pathological Subtypes	1.037	0.692–1.554	0.861	0.867	0.687–1.123	0.351
Pathological Grade	1.022	0.392–2.661	0.965	1.328	0.695–2.538	0.364
PFS exceeding 225 days	0.476	0.287–0.789	0.003			
monocyte ratio > 1	3.085	1.860–5.115	< 0.001	1.888	1.287–2.770	0.001
NLR ratio > 1	3.347	2.025–5.533	< 0.001	1.778	1.234–2.562	0.002

Similarly, no significant impact on PFS when analyzing with Age (*P* = 0.107), Sex (*P* = 0.150), size of primary tumor (*P* = 0.777), tumor depth (*P* = 0.120), pathological subtypes (*P* = 0.351) or pathological grade (*P* = 0.364) (Table 2). Median PFS was 225 days and the 1-year PFS rates were 33% (Fig. 2).

**Figure 3:**
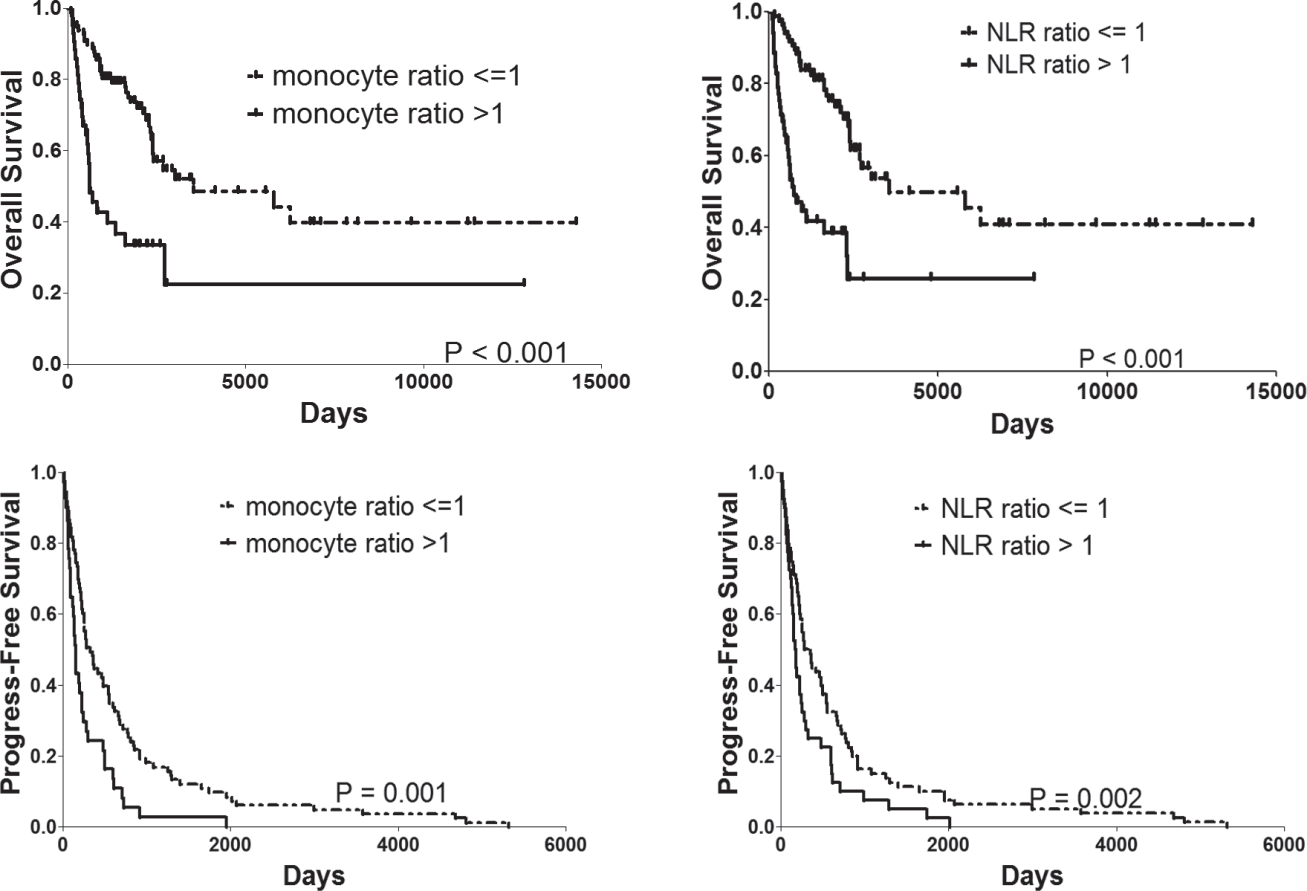
OS and PFS rate of patients with monocyte ratio > 1 vs. <= 1 and NLR ratio > 1 vs. <= 1. PFS progress-free survival, OS overall survival

OS was significantly worse in the monocyte ratio > 1 group (median OS 625 days) than the monocyte ratio <= 1 group (median OS 3544 days) (*P* < 0.001). Similar results could also be observed in the NLR ratio > 1 group (median OS 737 days) and NLR ratio <= 1 group (median OS 3544 days) (*P* < 0.001). Patients with monocyte ratio > 1 had a significantly worse PFS (median PFS 135 days) than those with monocyte ratio <= 1 (median PFS 274 days) (*P* = 0.001) and NLR ratio > 1 group (median PFS 141 days) and NLR ratio <=1 group (median PFS 283 days) (*P* = 0.002). The OS and PFS curves for the two groups are shown in Fig. 3.

Due to the reason that NLR is also an independent prognostic factor for some diseases, such as coronary artery disease (CAD), hypertension, diabetes and cerebrovascular disease. The effect of those diseases were evaluated on both NLR group and monocyte group (Supplemental Table S1).

**Figure 4:**
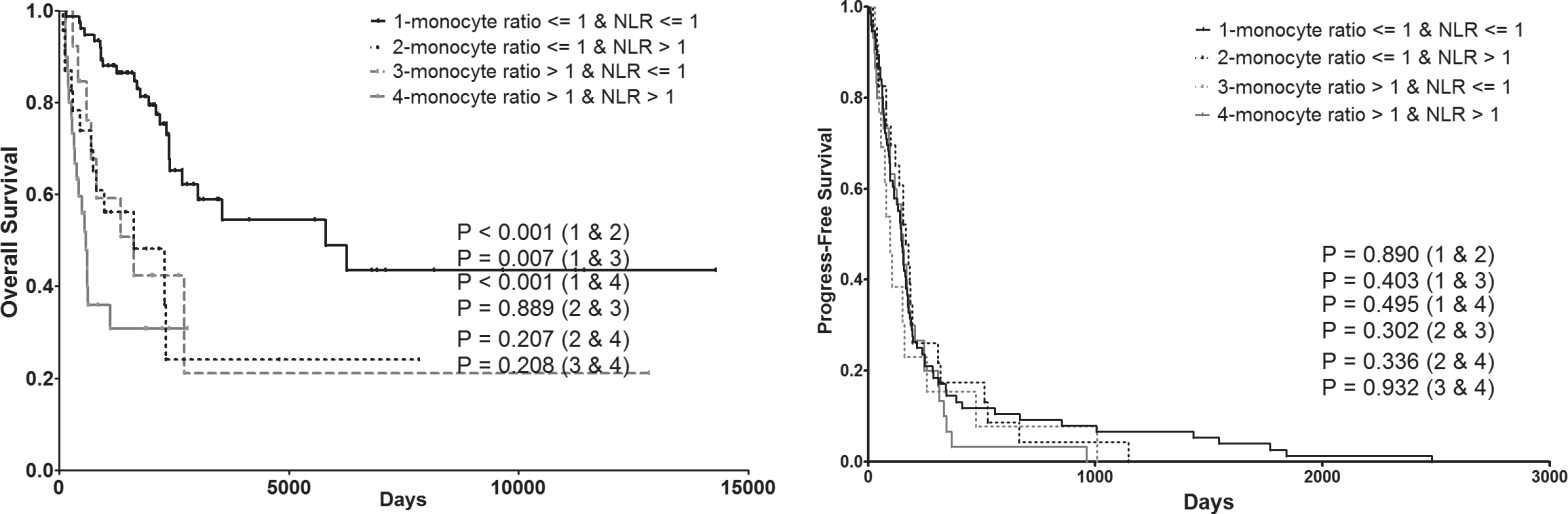
Combining prognostic value of monocyte ratio and NLR ratio on both OS and PFS. PFS progress-free survival, OS overall survival

Results of multivariable analysis to identify prognostic factors for OS and PFS in Metastatic STS patients are shown in Table 3. In multivariable analysis, the presence of NLR ratio > 1, and monocyte ratio > 1 were significantly associated with poor prognosis for both OS and PFS (monocyte ratio > 1, hazard ratio 1.999, 95% CI 1.141–3.504, *p* = 0.016; hazard ratio 1.628, 95% CI 1.080–2.455, *p* = 0.02, respectively) and (NLR ratio > 1, hazard ratio 2.477, 95% CI 1.423–4.311, *p* = 0.001; hazard ratio 1.531, 95% CI 1.035–2.265, *p* = 0.033, respectively). Patients were then divided into four categories according to different monocyte ratio and NLR ratio (group 1, monocyte ratio <= 1 and NLR ratio <= 1 (*n* = 76, 53.5%); group 2, monocyte ratio <= 1 and NLR ratio > 1 (*n* = 23, 16.2%); group 3, monocyte ratio > 1 and NLR ratio <= 1 (*n* = 13, 9.2%); and group 4, monocyte ratio > 1 and NLR ratio > 1 (*n* = 30, 21.1%)) to evaluate their combining prognostic value on both OS and PFS (Fig. 4). Accordingly, patients in group 1 showed significant better OS than patients in all the other 3 groups.

**Table 3:** Multivariable analysis of prognostic factors for OS and PFS in patients with Metastatic STS

Variables	OS			PFS		
	HR	95%CI	*P* value	HR	95%CI	*P* value
PFS exceeding 225 days	0.553	0.330–0.927	0.025			
monocyte ratio > 1	1.999	1.141–3.504	0.016	1.628	1.080–2.455	0.020
NLR ratio > 1	2.477	1.423–4.311	0.001	1.531	1.035–2.265	0.033

## DISCUSSION

This series of patients represents a single-institution nearly 50-year experience in the management of metastatic STS. As an important subgroup of STS [21, 22], metastatic STS still have an unsatisfying prognosis, despite of continuous treatment development [23–25]. Similar to localized disease, metastatic STS incorporates heterogeneous groups with different pathological subtypes or tumor sites, which result in broad prognostic spectrums. Previous studies reported little prognosis improvement of metastatic STS in last decades [23–25]. This is why we set out to determine whether possible risk factors, such as age, sex, primary tumor site, size of primary tumor, tumor depth, pathological subtypes, pathological grade, Monocyte ratio or NLR ratio, could predict either PFS or OS in patients with metastatic STS.

Publications have already shown that both the intrinsic characteristics and environment of tumor affected the invaded and metastatic ability [26]. Abnormal tumor phenotype may stimulate inflammatory cells flowing into tissues around the tumor. In addition, generalized and nonspecific inflammatory response could be triggered by generalized and nonspecific inflammatory response and following tissue destruction and disruption [27]. Evidence indicated an association among systemic inflammatory response, progressive nutritional and functional decline in cancer victims, and poor prognosis, which could be partly interpreted by insidious cancer progression activating innate immunity [10, 28].

A correlation between the increasing monocytes, neutrophils and decreasing lymphocytes and inflammation-induced tumor growth and progression via various growth and pro-angiogenic cytokines has been observed and proved, although immunosuppression is common in the cancer population [29–33].

Studies have showed peripheral blood monocytes to be an independent prognosis factor in patients with neck and head, biliary, cervix, liver, stomach and colon cancers [19, 31, 34–36]. Low blood neutrophil count and high lymphocyte count showed prognostic value in several studies [34, 37–39]. In addition, various evidences indicated elevating NLR accompanying poor prognosis in hepatocellular carcinoma, ovarian cancer, colorectal cancers, gastric cancer, and head and neck cancer [13, 16, 35, 40, 41]. Investigations revealed that this association might be caused by suppressed antitumor cellular immune activity of natural killer cells and lymphocyte by increased neutrophils [38]. Previous studies on circulating leukocyte influenced us concerning the association between clinical circulating monocytes, neutrophils, lymphocytes and tumor prognosis. Differently, relative large number of patients with metastatic STS were enrolled in current study. Furthermore, monocyte and NLR ratio, instead of cells counts, were used in our analysis, which could assess the impact of response to treatment.

Sarcomas, since at least as far back as 1891, have been suspected to have a relationship to the immune system [42]. Many sarcomas express highly immunogenic antigens, such as neo-antigens from specific translocations, cancer/testis antigens and microphthalmia transcription factor, and even the fusion proteins themselves, which could be seen as foreign by immune system and used as target of immunotherapy [43–45]. Over the past several years, various advances including tumor vaccination, adoptive cell transfer, biochemotherapy, and immune checkpoint blockade are rapidly emerging and benefiting sarcoma patients [46–48]. Preliminary result of dendritic cell vaccines suggested the tolerance and inducing immune response of tumor vaccination. Recombinant NY-ESO-1 intranodal injection before sirolimus indicated superior anti-tumor efficacy of mTOR inhibition through promoting CD8+ T-cell memory responses [49]. Takahashi treated 20 refractory sarcoma patients by employing 15 tumor-associated antigens with totally 31 peptides [50]. An objective clinical response in four of six patients with synovial cell sarcoma was obtained after adoptive transfer of autologous T-cells transduced with TCR against NY-ESO-1 [44]. One patient with epithelioid sarcoma was reported to be treated with expanded lymphocytes and natural killer cells [51]. Interleukin-2 therapy was used in angiosarcoma, although results of clinical trials were not yet available [52]. Ipilimumab, anti-CTLA-4 antibody, was halted for poor accrual in synovial sarcoma in a previous study [53]. Clinical trials of sarcoma-specific PD-1 targeted agents are still in the planning stages [54].

Several limitations remain in this study. First, all the data were retrospectively collected, thus clinical and survival comparison might be influenced by selection bias due to its retrospective nature. Second, a relatively small number of each histologic subtype was examined in this study, due to the reason that metastatic STS are extremely rare. It is substantial that the result of histologic subtypes not predicting PFS or OS might be caused by a Type II error. Although, we have enrolled sufficient number of metastatic STS to identify differences in other prognostic factors. Third, the monocyte phenotype or molecular information were not analyzed, which was cause by lack of these information in our retrospective data. Fourth, other systemic inflammatory immune index, such as C-reactive protein or albumin, which were known as prognostic factors [55], also absent in our retrospective data.

## MATERIALS AND METHODS

This study was approved by the institutional review board of Sun Yat-sen University Cancer Center (SYSUCC) and informed consent was obtained from each participant. Chart review was performed on 154 consecutive patients who suffered from STS with metastases between July 1965 and May 2013. Only patients with metastatic STS were included in current study, whereas those with osteosarcoma were not. Under these criteria, 142 of the 154 patients were enrolled in the final analysis. 12 patients with STS were excluded from analysis because of incomplete records. Characteristics of patients and tumors at initial diagnosis of STS and development of metastases were collected and tested for relationships with progress free survival (PFS) and overall survival (OS). The following factors were studied: patient age, sex (male vs. female), primary tumor size, and tumor depth (superficial vs. deep) at diagnosis. WHO classification [20] was used for determination of pathological diagnosis and tumor grade. All data were reviewed and confirmed by two independent pathologists.

Monocyte ratio was calculated as absolute monocyte count after initial treatment divided by absolute monocyte count before initial treatment. NLR was calculated as neutrophil count divided by lymphocyte count. NLR ratio was calculated as NLR after initial treatment divided by NLR before initial treatment. The blood tests were obtained within 24 hours for all patients as routine clinical practice in SYSUCC.

### Statistical analysis

The data are presented as the number (%) or median (range) unless otherwise stated. The Pearson χ2 test and Fisher's exact test were used for categorical data, and an independent sample *t*-test or the Mann–Whitney *U* test were used for numerical data.

PFS and OS curves were estimated using the Kaplan-Meier method. PFS was calculated from the date of initial diagnosis to the time of metastasis diagnosis, and OS from the date of initial diagnosis to the time of death reported. Risk factors of PFS and OS were assessed by univariate analysis with log rank test and multivariate analysis with Cox proportional hazards regression. Multivariate analysis was performed using Cox proportional hazard model. Cut-off value of PFS was established by the receiver operating characteristic (ROC) curve statistical analyses. All models for survival analyses were adjusted for age at diagnosis. *P* < 0.05 was considered to be significant in all statistical analyses. Data analysis was performed using SPSS 18.0 (PASW Statistics 18) for Windows (SPSS Inc, Chicago, IL).

## CONCLUSION

In current study, both monocyte ratio and NLR ratio were found to be significant prognostic predictors for OS and PFS of metastatic STS. Additionally, it was strongly believed that patients with monocyte ratio or NLR ratio > 1 should be screened out as candidates for more intensive or aggressive multimodality treatments and more aggressive follow-up. For this reason, this result could serve as a basis for future prospective study in selecting high risk patients for candidates for more aggressive multimodality treatments and more intensive follow-up and setting up a predicting model by subtyping the populations of monocytes or lymphocytes.

## SUPPLEMENTARY TABLE


