# Supplementary material for: Prognostic value of monocyte and neutrophils to lymphocytes ratio in patients with metastatic soft tissue sarcoma
Source: Oncotarget. 2015 Mar 20;6(11):9542–50. doi: 10.18632/oncotarget.3283 (PMC4496237; doi:10.18632/oncotarget.3283)
Supplement: Supplementary file 1 [file oncotarget-06-9542-s001.pdf]

SUPPLEMENTARY TABLE

Supplementary Table S1. Correlation of accompanied diseases with monocyte ratio and NLR rate and in Patients with Metastatic STS

Features	All (*n* = 142)	monocyte ratio <= 1 (*n* = 99)	monocyte ratio > 1 (*n* = 43)	*P*	NLR ratio <= 1 (*n* = 89)	NLR ratio > 1 (*n* = 53)	*P*
Coronary Artery Disease				0.999			0.293
Without	139	97	42		86	53	
With	3	2	1		3	0	
Hypertension				0.747			0.735
Without	127	88	39		79	48	
With	15	11	4		10	5	
Diabetes				0.811			0.499
Without	114	80	34		73	41	
With	28	19	9		16	12	
Cerebrovascular Disease				-			-
Without	142	99	43		89	53	
With	0	0	0		0	0	

-No statistics are calculated because no patients accompanied with Cerebrovascular Disease.
